# The Feasibility of Omega-3 Supplementation Compared to Placebo in the Management of Long COVID Symptoms Among Healthcare Workers: A Randomized Controlled Trial

**DOI:** 10.7759/cureus.76148

**Published:** 2024-12-21

**Authors:** Arunima Sarkar, Erin Speiser, Susan Dara, Chinwe Ogedegbe, Portia Chinnery, Marie-Therese Estanbouli, Lora Kasselman, Benjamin Kligler, Elli Gourna Paleoudis, Manisha Parulekar

**Affiliations:** 1 Geriatrics, Hackensack University Medical Center, Hackensack, USA; 2 The Deirdre Imus Environmental Health Center, Hackensack University Medical Center, Hackensack, USA; 3 Emergency Medicine, Hackensack University Medical Center, Hackensack, USA; 4 Patient Safety and Quality, Hackensack University Medical Center, Hackensack, USA; 5 Pharmacy and Clinical Services, Hackensack University Medical Center, Hackensack, USA; 6 Biostatistics, Hackensack University Medical Center, Hackensack, USA; 7 Office of Research Administration, Hackensack University Medical Center, Hackensack, USA

**Keywords:** covid-19, covid recovery, long covid, omega-3, pasc, postacute sequelae of covid-19

## Abstract

Background: COVID-19 is known to cause significant multisystem inflammatory responses, leading to symptoms beyond the acute phase of illness. These “long COVID" symptoms affect quality of life and interfere with daily activities. This pilot study looks at the feasibility, tolerability, and safety of omega-3 (docosahexaenoic acid+eicosapentaenoic acid, EPA) among healthcare workers with long COVID symptoms in New Jersey.

Methods: This double-blind, randomized-controlled pilot trial used self-administered omega-3 vs. placebo for 12 weeks in healthcare workers. The enrollment period was from October 2021 to March 2023. Participants were monitored weekly for compliance and adverse effects. They completed the Symptoms and Quality of Life survey biweekly. Baseline and week-12 blood test for omega-3 levels and arachidonic acid (AA):EPA ratio was also measured and analyzed. Descriptive statistics were calculated for all variables at 12 weeks. An independent sample t-test was conducted to compare the ages of the treatment groups. Fisher's exact tests were conducted on each outcome by the treatment arm. No adjustments for multiple testing were included; therefore, significance was set at p ≤ 0.05. Analyses were conducted using R version 4.3.3 (R Core Team, Vienna, Austria).

Results: Thirty-two healthcare workers were recruited, and 18 completed the study. Feasibility was assessed based on enrollment and compliance with the study protocol. There was no significant difference in age between the placebo and treatment groups. The intervention group did not show significant improvement in the long COVID symptoms: shortness of breath (p = 0.39), cough (p = 0.76), fatigue (p = 0.57), lack of taste (p = 0.10), and lack of smell (p = 0.10). In the placebo group, baseline average omega-3 and AA:EPA ratio were 4.09 (standard deviation, SD = 0.85) and 23.9 (SD = 13.4), respectively, and week-12 omega-3 and AA:EPA ratio were 4.46 (SD = 0.95) and 20.8 (SD = 6.0), respectively. For the supplement group, baseline average omega 3 and AA:EPA ratio were 3.75 (SD = 0.48) and 23.1 (SD = 8.3), respectively, and week-12 omega-3 and AA:EPA ratio were 5.97 (SD = 1.93) and 11.8 (SD = 14.0), respectively. One supplement-treated participant and five placebo-treated participants experienced adverse events. No serious adverse events were reported.

Conclusions: This pilot study successfully demonstrated the feasibility, safety, and tolerability of using omega-3 supplements for the treatment of long COVID syndrome. The study results did not show statistically significant improvement in the long COVID symptoms. The mean difference in the AA:EPA ratio in the placebo vs. supplement group showed a pronounced decline in inflammatory markers in the supplement group. However, our study did not show a connection between the decreased inflammatory markers and clinical symptoms. We may need a longer follow-up to understand the possible clinical benefits of the decreased AA:EPA ratio.

## Introduction

Following COVID-19 infection, a significant number of patients continue to experience a wide range of ongoing health problems lasting from several weeks to several months or longer [1]. Patients who suffer from after-effects for more than 12 weeks after contracting the virus meet the National Institutes of Health definition for “Post-acute Sequelae of COVID-19” or “long COVID syndrome” [1]. According to the Mayo Clinic, the most common signs and symptoms of long COVID syndrome include fatigue, shortness of breath (SOB), cough, joint pain, and chest pain [2]. Other long-term signs and symptoms may include muscle pain or headache, fast or pounding heartbeat, loss of smell or taste, cognition, concentration or sleep problems, and rash or hair loss. Additional symptoms include organ damage in the heart, lungs, and brain, blood clots, and blood vessel issues, as well as mood and fatigue issues. The long-term health effects of COVID-19 are continually being discovered, so the list of symptoms continues to be updated.

Even those initially recovering from a mild case of the virus may report long-term effects [2]. Effects may be most severe in patients with preexisting respiratory lung diseases such as asthma, chronic obstructive pulmonary disease, and/or who had a more severe form of COVID-19, including those hospitalized [3]. While studies are underway to determine the exact cause of this syndrome, the Center for Disease Control lists complications from a dysregulated inflammatory state as one of the possible causes [4].

The natural pathways in the human body for the cyclooxygenase (COX) and lipoxygenase (LOX) enzyme systems are to convert omega-6 arachidonic acid (AA) to proinflammatory eicosanoids and omega-3 eicosapentaenoic acid (EPA) to anti-inflammatory eicosanoids [5]. The competitive interaction between the COX/LOX enzymes and AA/EPA determines the intensity of the inflammatory attack. If the cellular EPA levels are higher than the usual range, it partly displaces AA and reduces the formation of proinflammatory cytokines, thereby decreasing inflammation [6,7]. The purpose of using omega-3 supplementation is to facilitate an increase in the cellular EPA levels to decrease the dysregulated inflammatory state and help in the recovery process, as EPA is also converted to proresolving mediators known as resolvins, protectins, and maresins. These mediators are responsible for reducing the magnitude and duration of inflammation, stimulating wound healing, and regenerating damaged tissue. A study conducted in Northern Spain assessed the relationship between post-COVID-19 syndrome and inflammatory markers and found mild elevations of certain biomarkers like C-reactive protein, which may be evidence of underlying low-grade inflammation in patients with long COVID syndrome [8]. Based on the assumption that long COVID syndrome is caused by a dysregulated inflammatory state and omega-3 supplements are known to have anti-inflammatory properties, we are interested in understanding its use and potential benefit in long COVID syndrome.

The primary objective of this study is to explore the feasibility of using omega-3 supplements vs. placebo among healthcare workers with long-term symptoms. This serves as a proof of concept for a future study to evaluate the efficacy of omega-3 supplementation among a larger population recovering from COVID-19.

The secondary objectives were to determine the safety/tolerability of the capsules and which symptoms of long COVID syndrome (if any) were positively affected by taking an omega-3 supplement. The long COVID symptoms included in this study are SOB, cough, fatigue, lack of taste, and lack of smell.

## Materials and methods

Trial design

This feasibility study was a double-blind, randomized controlled trial with two parallel treatment arms (1:1) conducted among Hackensack Meridian Health (HMH) healthcare workers in New Jersey. Results are presented here using the Consolidated Standards of Reporting Trials (CONSORT) 2010 extension to pilot and feasibility trial format. This study was registered on ClinicalTrials.gov with identifier number NCT05121766 as of November 16, 2021. The study was also registered with the FDA by IND #157250 (URL: https://classic.clinicaltrials.gov/ct2/show/NCT05121766?cond=COVID-19+Long+Covid&intr=Omega+3+fatty+acid&draw=2&rank=2).

Participants and sample size

This study was open to all 33,000 HMH team members across 13 network hospitals and approximately 500 outpatient locations. The enrollment period was from October 2021 to March 2023. For this feasibility study, 32 participants were recruited, 14 dropped out, and 18 completed the study. Three were male, and the rest were female participants.

Due to the pandemic, the study was conducted remotely, and the communication between the study participants and the research coordinator took place via email and telephone. The study was advertised widely across our network to increase diversity. Participants were asked to both self-report any side effects and respond to regular check-in calls. The screening was conducted to identify eligible participants from the pool of interested HMH employees who filled out the prescreening survey. The following inclusion/exclusion criteria were used for screening. Those who met the criteria were sent a consent form, followed by a baseline survey, and a participant unique ID was assigned.

Inclusion criteria

The inclusion criteria included the following: 1) 18 years and above, able to provide informed consent; 2) formal diagnosis of COVID-19 via polymerase chain reaction (PCR) test (if a home test was done, confirmed via PCR test); 3) outpatient treatment only for COVID-19; no hospitalization; 4) must be experiencing more than one of the following ongoing COVID-19 symptoms >12 weeks after initial infection: respiratory symptoms (SOB, cough), fatigue, loss of taste, and loss of smell; 5) symptom(s) coincided with COVID-19 infection and were not present prior to COVID-19 infection; 6) does not have a soy allergy or allergy to fish; 7) able to participate in biweekly surveys; 8) able to take blood pressure and record it in a biweekly survey; 9) not currently taking an omega-3 supplement or other high-dose supplement (over 2,000 international units, IU) with the potential for aiding the recovery of long COVID syndrome (e.g., zinc, vitamin C, elderberry), 10) able and willing to give a spot blood sample (two drops) at baseline and end of 12 weeks; and 11) able to take/swallow six mini-pills daily.

Exclusion criteria

The exclusion criteria included the following: 1) <18 years old, unwilling to provide informed consent/declined to take part; 2) no formal diagnosis of COVID-19 via PCR test; 3) hospitalized for the treatment of COVID-19; 4) not experiencing more than one ongoing COVID-19 symptoms >12 weeks being measured in this study: respiratory symptoms (SOB, cough), fatigue, loss of taste, and loss of smell; 5) symptom(s) did not coincide with COVID-19 infection and were present prior to COVID-19 infection; 6) does have a soy allergy and/or allergy to fish; 7) not able to participate in biweekly surveys; 8) not able to take own blood pressure and record it in the biweekly survey; 9) currently taking an omega-3 supplement or other high-dose supplement (over 2,000 IU) with the potential for aiding the recovery of long COVID syndrome (e.g., zinc, vitamin C, and elderberry), 10) not able and not willing to give a spot blood sample (two drops) at baseline and end of 12 weeks; and 11) unable to take/swallow six mini-pills daily.

Intervention

This double-blinded study consisted of two arms, and the participants were randomly assigned to either the intervention arm or placebo. Individual participants were shipped a 12-week supplement of either omega-3 (EPA+docosahexaenoic acid) or a matching placebo made from soybean oil. Both pills were identical in appearance. The dosage of the study drug (2,100 mg, six mini pills in two divided doses) was set following the guidelines from the American Heart Association and the U.S. Food and Drug Administration [9].

The study drug was self-administered twice daily for 12 weeks. The study required the participants to send blood samples to measure the omega-3 index levels at the baseline and at week 12. Participants had weekly check-in calls with the RC to report any adverse events, which were then reported to the HMH Data Safety Monitoring Board and the Food and Drug Administration (FDA). The participants completed a biweekly symptom and quality of life survey online in Research Electronic Data Capture (REDCap, Hackensack Meridian Health, Hackensack, NJ). The detailed description of the intervention is reported according to the Template for Intervention Description and Replication (Appendix A).

Randomization

The randomization assignments were generated via the Statistical Analysis System statistical software version 9.4, with a block size of 6 and a 1:1 ratio, and implemented by the research pharmacists, who were the unblinded party. The assignments were then linked to the REDCap database to merge prior to statistical summaries and analyses. All study team members and participants were blinded except for the Hackensack University Medical Center Hackensack Meridian Health network research pharmacists.

Statistical methods

Descriptive statistics (number, frequency, mean, median, standard deviation, SD, minimum, and maximum, as appropriate) were calculated for all variables at 12 weeks. An independent sample t-test was conducted to compare the age between the treatment groups. Fisher’s exact tests were conducted on each outcome by the treatment arm. No adjustments for multiple testing were included. Therefore, the significance was set at p ≤ 0.05. All analyses were conducted using R version 4.3.3 (R Core Team, Vienna, Austria, 2021).

## Results

Participant flow

**Figure 1:**
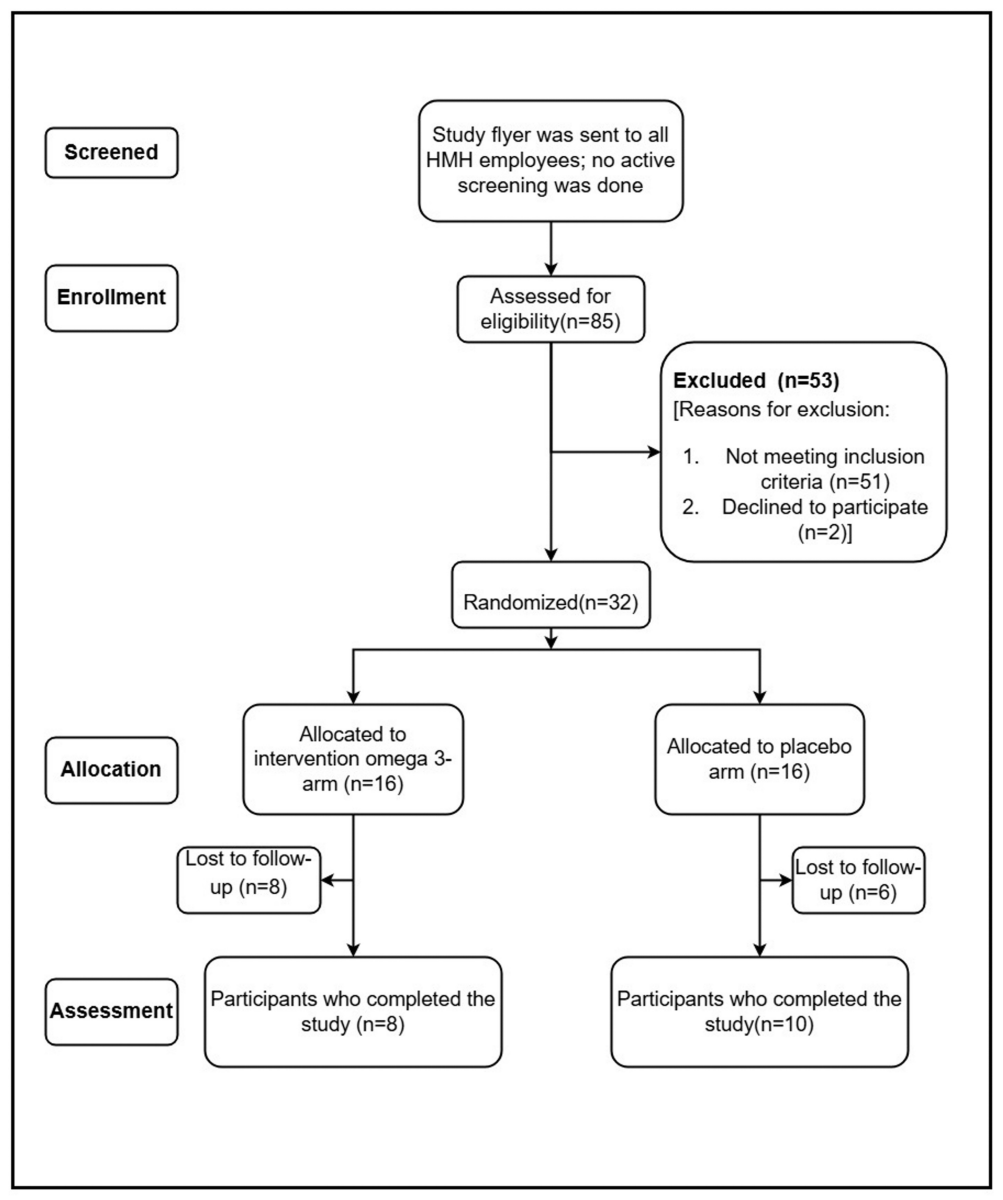
CONSORT extension for Pilot and Feasibility Trials Flow Diagram HMH: Hackensack Meridian Health; CONSORT: Consolidated Standards of Reporting Trials

Figure 1 shows the CONSORT extension for the pilot and feasibility trials flow diagram.

Recruitment

After Institutional Review Board approval, the study opened for enrollment in January 2022 and remained open for 12 months. Study flyers (see Appendix B) were sent out via internal communications (pulse newsletter) to HMH team members. The enrollment was hindered by changes in COVID treatment approaches, reinfections, and availability of vaccines.

Baseline data

The average age in the study was 50.1 years (SD = 11.9 years). There were 17 female (94.4%) and one male (5.6%) participants; 17 (94.4%) participants identified as White and two (11.1%) participants identified as Asian. Overall, three (16.7%) participants reported having diabetes, and two (11.1%) reported having chronic lung disease. There was no significant difference in age between placebo (50.3 years; SD = 13.2 years) and treatment groups (49.8 years; SD = 10.9 years; p = 0.92). Please refer to Table 1 for demographics and clinical characteristics.

**Table 1:** Demographics and clinical characteristics

Demographics and clinical characteristics	Placebo (n = 16)	Omega-3 supplement (n = 16)	Overall (n = 32)
Age			
Mean (SD)	47.3 (12.2)	46.4 (9.78)	46.8 (10.9)
Median (min, max)	50.5 (24, 66)	45 (27, 62)	47.5 (24, 66)
Gender, n (%)			
Female	14 (87.5%)	15 (93.8%)	29 (90.6%)
Male	2 (12.5%)	1 (6.3%)	3 (9.4%)
Black or African American, n (%)			
No	15 (93.8%)	15 (93.8%)	30 (93.8%)
Yes	1 (6.3%)	1 (6.3%)	2 (6.3%)
White, n (%)			
No	2 (12.5%)	1 (6.3%)	3 (9.4%)
Yes	14 (87.5%)	15 (93.8%)	29 (90.6%)
Asian, n (%)			
No	16 (100%)	15 (93.8%)	31 (96.9%)
Yes	0 (0%)	1 (6.3%)	1 (3.1%)
American Indian or Alaska Native, n (%)			
No	16 (100%)	16 (100%)	32 (100%)
Other, n (%)			
No	15 (93.8%)	15 (93.8%)	30 (93.8%)
Yes	1 (6.3%)	1 (6.3%)	2 (6.3%)
Unknown, n (%)			
No	16 (100%)	16 (100%)	32 (100%)
Ethnicity, n (%)			
Hispanic	2 (12.5%)	2 (12.5%)	4 (12.5%)
Non-Hispanic, n (%)	14 (87.5%)	14 (87.5%)	28 (87.5%)
Diabetes, n (%)			
No	15 (93.8%)	13 (81.3%)	28 (87.5%)
Yes	1 (6.3%)	3 (18.8%)	4 (12.5%)
Hypertension, n (%)			
No	12 (75%)	11 (68.8%)	23 (71.9%)
Yes	4 (25%)	5 (31.3%)	9 (28.1%)
High cholesterol, n (%)			
No	15 (93.8%)	10 (62.5%)	25 (78.1%)
Yes	1 (6.3%)	6 (37.5%)	7 (21.9%)
Stroke ischemic or hemorrhage, n (%)			
No	16 (100%)	16 (100%)	32 (100%)
Chronic lung diseases such as asthma, emphysema, or chronic bronchitis, n (%)			
No	13 (81.3%)	15 (93.8%)	28 (87.5%)
Yes	3 (18.8%)	1 (6.3%)	4 (12.5%)
Heart disease, n (%)			
No	15 (93.8%)	15 (93.8%)	30 (93.8%)
Yes	1 (6.3%)	1 (6.3%)	2 (6.3%)
Cancer (other than skin cancer), n (%)			
No	13 (81.3%)	15 (93.8%)	28 (87.5%)
Yes	3 (18.8%)	1 (6.3%)	4 (12.5%)
Depression, n (%)			
No	11 (68.8%)	11 (68.8%)	22 (68.8%)
Yes	5 (31.3%)	5 (31.3%)	10 (31.3%)
Human immunodeficiency virus/acquired immunodeficiency syndrome, n (%)			
No	16 (100%)	16 (100%)	32 (100%)
Chronic obstructive pulmonary disease, n (%)			
No	16 (100%)	16 (100%)	32 (100%)
Other, n (%)			
No	12 (75%)	14 (87.5%)	26 (81.3%)
Yes	4 (25%)	2 (12.5%)	6 (18.8%)
Smoking, n (%)			
No, but I used tobacco products in the past	3 (18.8%)	7 (43.8%)	10 (31.3%)
No, I have never used tobacco products	10 (62.5%)	9 (56.3%)	19 (59.4%)
Yes, I currently use tobacco products	3 (18.8%)	0 (0%)	3 (9.4%)
Have you received the COVID-19 vaccine? n (%)			
Not answered	1 (6.3%)	0 (0%)	1 (3.1%)
No	1 (6.3%)	2 (12.5%)	3 (9.4%)
Yes	14 (87.5%)	14 (87.5%)	28 (87.5%)
Are you currently experiencing any side effects from the COVID-19 vaccine? n (%)			
Not answered	2 (12.5%)	2 (12.5%)	4 (12.5%)
No	12 (75%)	14 (87.5%)	26 (81.3%)
Yes	2 (12.5%)	0 (0%)	2 (6.3%)

Numbers analyzed, outcomes, and estimation

The study screened 85 participants; 51 met the eligibility criteria, 32 participants consented, and 18 completed the study. Seven out of 16 subjects in the placebo group and six out of 16 in the supplement group completed both baseline and week-12 blood tests. One omega-3 supplement-treated participant and five placebo-treated (soybean oil) participants experienced adverse events. Four participants from the placebo group dropped out of the study because of the side effects of the capsule. No serious adverse events were reported in either group.

The intervention group did not show significant improvement in the long COVID symptoms: SOB (OR = 0.44, 95% CI = 0.06-2.61, p = 0.39), cough (OR = 0.75, 95% CI = 0.12-4.56, p = 0.76), fatigue (OR = 1.92, 95% CI = 0.22-20.14, p = 0.57), lack of taste (OR = 6.85, 95% CI = 0.98-71.97, p = 0.10), lack of smell (OR = 6.85, 95% CI = 0.98-71.97, p = 0.10) (Table 2).

**Table 2:** Outcomes (excluding those lost to follow-up) at 12 weeks by treatment arm ^*^Independent samples t-test or Fisher's exact test

Outcomes at 12 weeks	Placebo (n = 10)	Omega-3 supplement (n = 8)	Overall (n = 18)	p value^*^
Age				
Mean (SD)	50.3 (13.2)	49.8 (10.9)	50.1 (11.9)	0.9241
Median (min, max)	55.5 (24, 66)	52.5 (36, 62)	54.5 (24, 66)	
Shortness of breath, n (%)				
N/A: I never experienced this symptom	1 (10%)	2 (25%)	3 (16.7%)	0.8272
During the first two weeks since my COVID-19 diagnosis	2 (20%)	1 (12.5%)	3 (16.7%)	
During the third week since my COVID-19 diagnosis	0 (0%)	1 (12.5%)	1 (5.6%)	
During the fourth week since my COVID-19 diagnosis	2 (20%)	1 (12.5%)	3 (16.7%)	
I still experience this symptom	5 (50%)	3 (37.5%)	8 (44.4%)	
Cough, n (%)				
N/A: I never experienced this symptom	0 (0%)	0 (0%)	0 (0%)	0.1955
During the first two weeks since my COVID-19 diagnosis	2 (20%)	0 (0%)	2 (11.1%)	
During the third week since my COVID-19 diagnosis	0 (0%)	3 (37.5%)	3 (16.7%)	
During the fourth week since my COVID-19 diagnosis	5 (50%)	2 (25%)	7 (38.9%)	
I still experience this symptom	3 (30%)	2 (25%)	5 (27.8%)	
Missing	0 (0%)	1 (12.5%)	1 (5.6%)	
Fatigue, n (%)				
N/A: I never experienced this symptom	0 (0%)	0 (0%)	0 (0%)	1.0000
During the first two weeks since my COVID-19 diagnosis	1 (10%)	0 (0%)	1 (5.6%)	
During the third week since my COVID-19 diagnosis	0 (0%)	0 (0%)	0 (0%)	
During the fourth week since my COVID-19 diagnosis	3 (30%)	2 (25%)	5 (27.8%)	
I still experience this symptom	5 (50%)	5 (62.5%)	10 (55.6%)	
Missing	1 (10%)	1 (12.5%)	2 (11.1%)	
Lack of taste, n (%)				
N/A: I never experienced this symptom	5 (50%)	1 (12.5%)	6 (33.3%)	0.4159
During the first two weeks since my COVID-19 diagnosis	1 (10%)	1 (12.5%)	2 (11.1%)	
During the third week since my COVID-19 diagnosis	1 (10%)	1 (12.5%)	2 (11.1%)	
During the fourth week since my COVID-19 diagnosis	1 (10%)	0 (0%)	1 (5.6%)	
I still experience this symptom	2 (20%)	4 (50%)	6 (33.3%)	
Missing	0 (0%)	1 (12.5%)	1 (5.6%)	
Lack of smell, n (%)				
N/A: I never experienced this symptom	5 (50%)	1 (12.5%)	6 (33.3%)	0.4159
During the first two weeks since my COVID-19 diagnosis	1 (10%)	1 (12.5%)	2 (11.1%)	
During the third week since my COVID-19 diagnosis	1 (10%)	1 (12.5%)	2 (11.1%)	
During the fourth week since my COVID-19 diagnosis	1 (10%)	0 (0%)	1 (5.6%)	
I still experience this symptom	2 (20%)	4 (50%)	6 (33.3%)	
Missing	0 (0%)	1 (12.5%)	1 (5.6%)	

In the placebo group, baseline average omega-3 and AA:EPA ratio were 4.09 (SD = 0.85) and 23.9 (SD = 13.4), respectively, and week-12 omega-3 and AA:EPA ratio were 4.46 (SD = 0.95) and 20.8 (SD = 6.0), respectively. For the supplement group, baseline average omega-3 and AA:EPA ratio were 3.75 (SD = 0.48) and 23.1 (SD = 8.3), respectively, and week-12 omega-3 and AA:EPA ratio were 5.97 (SD = 1.93) and 11.8 (SD = 14.0), respectively (Table 3).

**Table 3:** Baseline and week-12 blood omega-3 levels and inflammatory marker (AA:EPA ratio) among study participants AA: arachidonic acid; EPA: eicosapentaenoic acid

Blood omega-3 and inflammatory marker (AA:EPA) levels	Placebo		Omega-3 supplement	
	Baseline (n = 7)	Week 12 (n = 7)	Baseline (n = 6)	Week 12 (n = 6)
Omega-3 index				
Mean (SD)	4.09 (0.846)	4.46 (0.947)	3.75 (0.483)	5.97 (1.93)
Median (min, max)	4.31 (2.68, 5.17)	4.46 (2.75, 5.58)	3.82 (2.93, 4.21)	5.82 (3.49, 8.88)
AA:EPA ratio				
Mean (SD)	23.9 (13.4)	20.8 (5.96)	23.1 (8.26)	11.8 (14)
Median (min, max)	20.7 (11.1, 47.8)	20.6 (13.4, 29.4)	22.2 (14.3, 37.8)	5.05 (2.30, 38.7)
Difference in baseline and week-12 blood omega-3 levels				
Mean (SD)	-	-0.373 (0.551)	-	-2.23 (1.66)
Median (min, max)	-	-0.0700 (-1.14, 0.140)	-	-2.18 (-4.72, 0.150)
Missing	-	0 (0%)	-	0 (0%)
Difference in baseline and week-12 blood AA:EPA ratio				
Mean (SD)	-	3.06 (9.77)	-	11.4 (19.7)
Median (min, max)	-	-1.80 (-5.60, 18.4)	-	18.2 (-21.7, 33.4)
Missing	-	0 (0%)	-	0 (0%)

Harms (adverse events)

There were no serious adverse events. In the supplement group, one out of 16 participants experienced adverse events; in the placebo group, five out of 16 experienced adverse events. All adverse events were reported to the FDA.

The participant receiving the supplement reported worsening fatigue. Four participants in the placebo group reported rash, gastrointestinal side effects (nausea, heartburn, and diarrhea), and bleeding (oral, nose, and lower gastrointestinal bleeding). One person from the placebo group had sharp pain on the left side of the chest (breastbone area under the armpit) and reported experiencing similar pain in the past when the participant was diagnosed with pleurisy.

## Discussion

This pilot study aimed to assess the feasibility of serving as a proof of concept for future studies evaluating the efficacy of omega-3 supplementation and determining the safety/tolerability of the placebo and omega-3 supplement. The results of this study support the feasibility and tolerability of utilizing omega-3 supplements in patients with long COVID symptoms. Participants tolerated the supplement well, the supplement arm had fewer adverse events than the placebo arm, and the reactions were not serious.

Omega-3, an over-the-counter nutritional supplement, is known to have an anti-inflammatory response, reducing the risk and severity of infection, and has been shown to reduce inflammatory markers among patients suffering from severe COVID-19 infection [10-13]. This study was designed based on the evidence that omega-3 supplements reduce the dysregulated inflammatory state, one of the possible etiologies for long COVID syndrome [4]. The study analyzed five commonly known long COVID symptoms: SOB, cough, fatigue, lack of taste, and lack of smell; laboratory blood omega-3 index values; and AA: EPA ratio (inflammatory markers) at baseline and week 12.

The week-12 outcome analysis of clinical symptoms for each symptom was done between the placebo and the omega-3 intervention groups. The percentage of participants in the placebo group who still experienced SOB, cough, fatigue, lack of taste, and lack of smell were about 50%, 30%, 50%, 20%, and 20%, respectively. In the supplement group, the percentage of participants still experiencing SOB, cough, fatigue, lack of taste, and lack of smell were about 50%, 37.5%, 25%, 50%, and 50%, respectively.

The study participants' baseline and week-12 blood omega-3 index values [14] and AA:EPA ratios [6] were assessed separately. A lower AA:EPA ratio represents higher inflammatory levels and is used as a marker of chronic inflammation [6]. The omega-3 levels in the placebo group had a mean difference of -0.37, whereas the supplement had a mean difference of -2.22. The mean difference in the AA:EPA ratio in the placebo arm was 3.06, whereas the supplement group had 11.4, indicating a more pronounced decline in inflammatory markers in the supplement group.

However, the study results did not show statistically significant improvement in the long COVID symptoms. Our sample size was significantly small and insufficient to demonstrate the efficacy of the omega-3 supplement. Our study did not show a connection between the decreased AA:EPA ratio and clinical symptoms. We may need a longer follow-up to understand the possible clinical benefits of the decreased AA:EPA ratio.

Limitations

Due to the pandemic's continually evolving landscape, such as the availability of the COVID-19 vaccine and prospective participants' proactive pretrial omega-3 supplement intake, recruiting and retaining participants for the study was challenging. Recurrent COVID-19 infection was another limiting factor for recruitment. The study did not consider screening for an omega-3-rich diet, antihistamine, guanfacine, low-dose naltrexone, and COVID-19 vaccination status among potential participants. The study did not monitor meal patterns and food frequency. Four of the participants from the placebo and one from the omega-3 group received COVID-19 vaccine during the study.

The study had to be conducted remotely due to the pandemic; however, a statistically significant sample size could be achieved if the study was conducted in person and included a larger, diverse population.

Generalizability

Our study only included healthcare workers, and therefore, future studies should include a more diverse study population. However, the methods can be used in a future, larger, and more definitive trial to test omega-3 supplements for the treatment of long COVID syndrome.

## Conclusions

This pilot study successfully demonstrated the feasibility, safety, and tolerability of using omega-3 supplements for the treatment of long COVID syndrome. The results did not show statistically significant improvement in the long COVID symptoms. The mean difference in the AA:EPA ratio in the placebo vs. supplement group showed a pronounced decline in inflammatory markers in the supplement group. However, our study did not show a connection between the decreased inflammatory markers and clinical symptoms. We may need a longer follow-up to understand the possible clinical benefits of the decreased AA:EPA ratio.

## Figures and Tables

**Figure 2:**
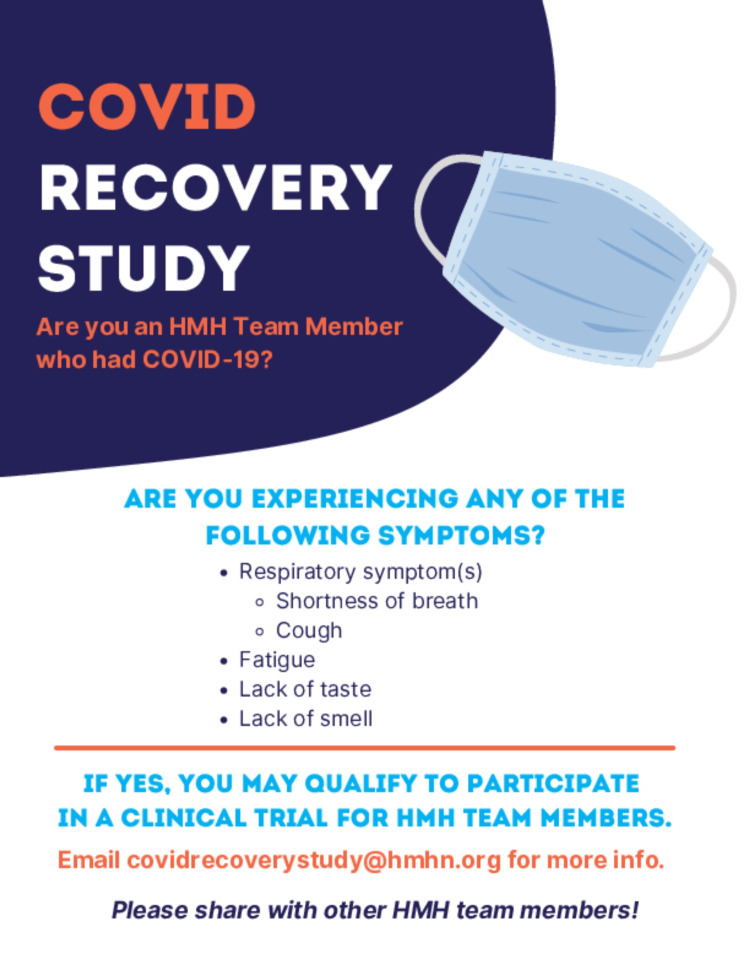
Flyer

## Appendices

Appendix A

**Table 4:** Template for intervention description and replication CDC: Centers for Disease Control and Prevention; COX: cyclooxygenase; DHA: eicosapentaenoic acid; EPA: eicosapentaenoic acid; FDA: Food and Drug Administration; HMH: Hackensack Meridian Health; LOX: lipoxygenase; RC: research coordinator; REDCap: Research Electronic Data Capture

Item	Description
Brief name: provide the name or a phrase that describes the intervention	Omega-3 (EPA+DHA) supplement, 2,100 mg, six mini pills/day vs. placebo (made from soybean oil)
Why: describe any rationale, theory, or goal of the elements essential to the intervention	CDC lists a dysregulated inflammatory state as one of the possible causes of long COVID syndrome. The natural pathways of the COX and LOX enzyme systems produce proinflammatory and anti-inflammatory eicosanoids, which help reduce inflammation and promote healing. The rationale for using omega-3 supplements is to facilitate an increase in the cellular EPA levels to decrease the dysregulated inflammatory state
What (materials): describe any physical or informational materials used in the intervention, including those provided to participants or used in intervention delivery or in training of intervention providers. Provide information on where the materials can be accessed (for example, online appendix, URL)	Study Flyer (Appendix B)
What (procedures): describe each of the procedures, activities, and/or processes used in the intervention, including any enabling or support activities	Research procedures: participants who contacted the study team at the address on the flier received an eligibility survey via REDCap to determine if they met the inclusion criteria. They were assigned an ID# in REDCap. Participants who met the inclusion criteria received a consent form via REDCap and were encouraged to contact the study team with questions via phone call or email. Once they consented, a demographic survey was sent to the participants. The participant was randomly assigned to receive either omega-3 (study drug) or soybean oil (placebo) capsules. A package containing the drug/placebo + two spot blood sample test kits for omega-3 levels was shipped to the study subject’s home. Study participants received two shipments in total during the course of the study. Shipment 1 upon enrolment and shipment 2 after week 4. The study participants in each arm were required to give a spot sample of blood at baseline and again after 12 weeks. The subject checked their blood pressure and recorded it in the REDCap biweekly survey. Side effects and adverse events were documented in REDCap by the RC and reported as required to HMH and/or FDA. The final study activity included the second spot blood sample and a short survey (the survey link was emailed to the subject via REDCap) to collect feasibility and acceptability data. The subject received a $40 gift card via mail after study participation. RC informed participants of their omega-3 level after lab results were returned and the subject’s participation in the study was complete. Monitoring of participants: the RC called each subject weekly to ensure procedures were being followed. The participants were encouraged to ask any questions during the call or contact the RC anytime. Participants in both arms were tracked for 12 weeks. For the first 17 participants, an interim analysis was done to determine whether any arm should be adjusted or terminated. The participants continued to receive standard care and follow-up for any routine conditions being managed at Hackensack Meridian Health
Who provided: for each category of intervention provider (for example, psychologist and nursing assistant), describe their expertise, background, and any specific training given	The intervention did not include any medical procedures
How: describe the modes of delivery (such as face-to-face or by some other mechanism, such as internet or telephone) of the intervention and whether it was provided individually or in a group	Each study subject received the study drug/placebo along with instructions on how to take the drug. For 12 weeks, the RC had a weekly “check-in call” with each study subject
Where: describe the type(s) of location(s) where the intervention occurred, including any necessary infrastructure or relevant features	The study drug was self-administered by the patient at home. The study supplies, including the study drug/placebo, were mailed to the participant’s home
When and how much: describe the number of times the intervention was delivered and over what period of time, including the number of sessions, their schedule, and their duration, intensity, or dose	The investigational drug omega-3 (EPA+DHA) is dosed at 2,100 mg per day via three mini capsules, two times/day (a total of six mini capsules per day). Each capsule has 252 mg of EPA and 102 mg of DHA. Support for dosing: the American Heart Association says taking up to 3 g of fish oil daily in supplement form is considered safe. Up to 5,000 mg of omega-3 fatty acids daily is considered safe. The U.S. FDA recommends consuming no more than 3 g/day of EPA and DHA combined, including up to 2 g/day from dietary supplements. Arm 2: placebo made from soybean oil (the same dosing schedule as the intervention arm) supplement and placebo were supplied by KD Pharma (Switzerland). The supplement was OceanBlue Omega-3 EPA+DHA (KD Pharma)
Tailoring: if the intervention was planned to be personalized, titrated, or adapted, then describe what, why, when, and how	No tailoring was done
Modifications: if the intervention was modified during the course of the study, describe the changes (what, why, when, and how)	No modification to the intervention was done
How well (planned): if intervention adherence or fidelity was assessed, describe how and by whom; and if any strategies were used to maintain or improve fidelity, describe them	Intervention adherence was not assessed
How well (actual): if intervention adherence or fidelity was assessed, describe the extent to which the intervention was delivered as planned	Intervention adherence was not assessed

Appendix B
